# Nebulized Ketamine Used for Managing Ankle Fracture in the Prehospital Emergency Setting: A Case Report

**DOI:** 10.5811/cpcem.2023.1.58761

**Published:** 2023-02-09

**Authors:** Eric Quinn, Sean Dhanraj, Joseph Liu, Sergey Motov, Matt Friedman, David Eng

**Affiliations:** Maimonides Medical Center, Department of Emergency Medicine, Brooklyn, New York

**Keywords:** prehospital care, non-opioid analgesia, emergency medical services, nebulized ketamine, case report

## Abstract

**Introduction:**

Acute traumatic limb injury is a common complaint of patients presenting to the emergency department (ED). Ketamine is an effective analgesic administered via intravenous (IV), intranasal (IN), intramuscular (IM), and nebulized routes in the ED. It has also been used in the prehospital setting via IV, IM, and IN routes. Recent studies have proposed the prehospital use of nebulized ketamine via breath-actuated nebulizer (BAN) as a noninvasive and effective method of analgesic delivery, as well as an alternative to opioid analgesia.

**Case Report:**

We present a case of a patient with right ankle fracture after a 12-foot fall who subsequently received 0.75 milligrams per kilogram of nebulized ketamine via BAN in the prehospital setting. The patient reported improvement of pain from 8/10 to 3/10 on the pain scale without need for additional pain medication during prehospital transport. This report supports the use of nebulized ketamine via BAN in the prehospital setting for acute traumatic limb injuries.

**Conclusion:**

The use of nebulized ketamine via BAN in the prehospital setting may be an effective analgesic option for the management of patients with acute traumatic limb injuries, particularly in those with difficult IV access, where mucosal atomization devices are not accessible, or where opioid-sparing treatments are preferable.

## INTRODUCTION

The N-methyl-D-aspartate/glutamate receptor complex antagonist ketamine decreases pain by diminishing central sensitization and hyperalgesia.^1^ Ketamine produces anesthesia in which the limbic system is selectively anesthetized. Sensory stimuli are, therefore, prevented from reaching the cerebral cortex, producing analgesia. Ketamine is an effective analgesic administered via intravenous (IV), intranasal (IN), intramuscular (IM), and nebulized routes in the emergency department (ED). Nebulized ketamine via breath-actuated nebulizer (BAN) has been more recently studied in the ED for pain control.^2^–^4^

Breath-actuated nebulizer is a specific nebulizer that creates aerosol only when a patient is inhaling, rather than creating aerosol continuously. In the prehospital setting, ketamine has been used widely and safely in a variety of indications, such as agitation, analgesia, and intubation traditionally via IV, IM, and IN routes.^5^–^8^ This report details the use of nebulized ketamine via BAN in the prehospital setting for acute traumatic limb injuries as a noninvasive and effective method of analgesic delivery, as well as an alternative to opioid analgesia.

## CASE REPORT

A 38-year-old male with no past medical history asked a bystander to call emergency medical services (EMS) after he had fallen 12 feet off a fire escape and landed on his right ankle. An Advanced Life Support ambulance was dispatched and, upon arrival, paramedics found the patient on the ground and unable to ambulate. He complained of 8/10 pain to his right ankle. Airway, breathing, and circulation to all four extremities were intact, and initial vital signs were heart rate (HR) of 100 beats per minute, blood pressure (BP) of 142/76 millimeters of mercury (mm Hg), respiratory rate (RR) of 20 breaths per minute, and oxygen saturation (SpO_2_) of 98%. Physical exam was notable for a right ankle deformity.

Paramedics on scene immobilized the patient’s leg in a splint, after which they contacted their online medical control (OLMC) center to discuss options for analgesia. The OLMC physician authorized the delivery of a dose of nebulized ketamine via BAN at 0.75 milligrams per kilogram (mg/kg) mixed with three milliliters (mL) of normal saline. During administration, the patient reported dizziness, which was rated one on a scale from zero to four using the Side Effects Rating Scale of Dissociative Anesthetics. This is a scoring system used to grade severity of medication side effects where zero represents “no change” in symptoms and four represents “very bothersome” symptoms.^9^ He did not become agitated, dissociated, or sedated.

Thirty minutes after the start of nebulization, pain was measured at 3/10. Repeat vitals at this time were HR 103 beats per minute, BP 128/79 mm Hg, RR 18 breaths per minute, and SpO_2_ 97%. In the ED, at one hour after administration of nebulized ketamine, the patient reported a return of his pain, and was given a dose of four mg IV morphine. Radiograph revealed a right tibial pilon fracture. He was placed in a posterior short leg splint and admitted to the orthopedic service; he was operated on the next day for open reduction and internal fixation of the right ankle joint. He was subsequently discharged post-procedure with orthopedic follow-up.

## DISCUSSION

Nebulized ketamine via BAN was successfully used to deliver timely analgesia in a patient with acute traumatic limb injury. At the time of this case, regional EMS protocols permitted use of narcotic medications without prior physician authorization only in the case of isolated extremity injuries. Although there did not appear to be signs of injury aside from the obvious right ankle deformity, the paramedics in this case were concerned for the possibility of additional occult injury based on the injury mechanism. They therefore opted to discuss the case with OLMC prior to treatment; the order for BAN ketamine then came at the discretion of the OLMC physician over the phone.

*CPC-EM Capsule*

What do we already know about this clinical entity?

*Low dose ketamine is a well-established medication for pain control via the intravenous route and its use has been explored via the inhalation route.*

What makes this presentation of disease reportable?

*Although there are instances of nebulized ketamine use in the emergency department, there are very few cases of nebulized ketamine in the prehospital setting.*

What is the major learning point?

*Nebulized tamine via breath-actuated nebulizer is feasible in the prehospital setting and can produce effective pain control with minimal side effects.*

How might this improve emergency medicine practice?

*Early non-opioid pain control may be achievable in the prehospital setting without need for intravenous access.*

The paramedics in this case reported minimal side effects and no significant changes in the vital signs while administering BAN ketamine. Additionally, they stated they would use this approach in the future if permitted because they were able to deliver pain relief quickly prior to obtaining eventual IV access. This is consistent with a national survey of paramedics, where 94% of paramedics who administered ketamine reported that they would use it again if given the opportunity.^10^ This patient showed a decrease in pain by 57.5% and was provided relief for one hour. This was enough time for the patient to be transported to the ED, complete ED registration and triage processes, and be assigned an ED nurse who could then provide additional analgesics. The patient was very satisfied with the BAN ketamine given by EMS and requested a second dose of it in the ED. By that point, IV access had been established and he was instead given IV analgesics by the primary ED care team for more prolonged pain relief.

Nebulized ketamine via BAN uses a compressed air source to convert liquid medication into an aerosol to allow for inhalation, producing smaller particles and greater dose delivery efficiency than standard continuous nebulizers. A BAN has two modes: one that functions as a standard continuous nebulizer, and one that is patient triggered. One study showed a reduction in ambient medication loss by greater than 85% with the use of BAN in comparison to standard continuous nebulizers, providing greater medication delivery to patients and reduced risks of ambient exposure to healthcare staff.^11^ [We have no financial relationships with any BAN device manufacturer.]

This case report supports past literature on the use of nebulized ketamine in the prehospital setting for acute traumatic limb injuries. A recent study on the use of nebulized ketamine vs IV morphine in the prehospital setting found no difference in the level of pain control achieved by either treatment, with decreased rates of side effects, such as nausea or vomiting, in those treated with nebulized ketamine.^12^ With a lower side effect profile in comparison to opioids, nebulized ketamine may be an effective alternative to opioid analgesia. Ketamine inhalation in healthy volunteers is easily tolerated and not associated with oropharyngeal irritation, hypersalivation, laryngospasm, cough, dyspnea, tachypnea, aspiration, cardiac dysrhythmias, or desaturations.^13^,^14^

Ketamine should be used with caution in those patients who have schizophrenia, are pregnant, or are allergic to the medication. Respiratory side effects can be readily managed by ventilatory support via bag-valve-mask, a skill all prehospital personnel are trained in. Another common side effect is nausea or vomiting, which can be managed with anti-emetics such as ondansetron which ALS can administer. Lastly, agitation is an uncommon side effect that is also within the scope of ALS practice to manage, with techniques ranging from verbal de-escalation to IM antipsychotics.

A dosage of 0.75 mg/kg of ketamine along with three mL of normal saline was administered in the prehospital setting. This dosing comes from the anesthesia literature for post-intubation sore throat, where the average decrease of postoperative throat pain was 44–50% without any major side effects.^15^ Furthermore, previous studies comparing ketamine dosed at 0.75 mg/kg, 1 mg/kg, and 1.5 mg/kg showed similar efficacy in reduction of pain and side-effect profile.^3^ While patients can receive up to 1.5 mg/kg, in this particular case, pain was well-controlled and reduced by 57.5% after 0.75 mg/kg.

In situations when IV access is not readily available or where mucosal atomization devices are not easily accessible for IN administration, the use of nebulized ketamine via BAN should be considered for delivery of timely analgesia. This is particularly advantageous in the prehospital setting, in which personnel, time, and resources may be scarce and placing IV access in an ambulance is often difficult. In our region, Basic Life Support ambulances staffed by emergency medical technicians (EMT) often respond to patients with traumatic injuries but have limited options to treat pain in these cases. While EMTs are trained in administering medications by nebulization (e.g., for asthma or chronic obstructive pulmonary disease exacerbations) they are not trained in obtaining IV access or administering IV medications. Use of nebulized analgesics such as ketamine would expand the ability of prehospital personnel to better care for patients in pain. Implementation may help to improve patient satisfaction scores with regard to better pain control, optimize unit-hour utilization with less time spent on moving patients with painful injuries, and decrease risk of employee needle-stick injuries associated with obtaining IV access.

## CONCLUSION

The use of nebulized ketamine via BAN in the prehospital setting may be an effective analgesic option for the management of patients with acute traumatic limb injuries, particularly in those with difficult IV access, where mucosal atomization devices are not accessible, or where opioid-sparing treatments are preferable. The implementation of protocols regarding BAN ketamine in the prehospital setting would provide EMS personnel with an additional pain control modality for use in the out-of-hospital setting. The ideal patient is one with acute musculoskeletal pain, but the use of BAN ketamine could be expanded in the future to include other chief complaints, such as burns.

Further studies are needed to continue to evaluate the safety and efficacy of nebulized ketamine in the prehospital setting. Possible confounders include variability in how well EMS personnel immobilize, extricate, and transport patients given their specific injury. With the increased interest in non-opioid treatment and interest in improving the overall patient experience from prehospital to discharge, further prospective clinical trials are warranted to evaluate patient satisfaction, potential for decreased opioid requirements in the ED, and a shorter time to disposition.

## References

[1] Gao M, Rejaei D, Liu H (2016). Ketamine use in current clinical practice. Acta Pharmacol Sin.

[2] Drapkin J, Masoudi A, Butt M (2020). Administration of nebulized ketamine for managing acute pain in the emergency department: a case series. Clin Pract Cases Emerg Med.

[3] Dove D, Fassassi C, Davis A (2021). Comparison of nebulized ketamine at three different dosing regimens for treating painful conditions in the emergency department: a prospective, randomized, double-blind clinical trial. Ann Emerg Med.

[4] Rhodes AJ, Fagan MJ, Motov SM (2021). Nebulized ketamine for managing acute pain in the pediatric emergency department: a case series. Turk J Emerg Med.

[5] Svenson JE, Abernathy MK (2007). Ketamine for prehospital use: new look at an old drug. Am J Emerg Med.

[6] Porter K (2004). Ketamine in prehospital care. Emerg Med J.

[7] Moy RJ, Le Clerc S (2011). Ketamine in prehospital analgesia and anaesthesia. Trends Anaesth Crit Care.

[8] Andolfatto G, Innes K, Dick W (2019). Prehospital analgesia with intranasal ketamine (pain-k): a randomized double-blind trial in adults. Ann Emerg Med.

[9] Eide PK, Stubhaug A, Øye I (1995). Continuous subcutaneous administration of the N-methyl-D-aspartic acid (NMDA) receptor antagonist ketamine in the treatment of post-herpetic neuralgia. Pain.

[10] Buckland DM, Crowe RP, Cash RE (2018). Ketamine in the prehospital environment: a national survey of paramedics in the United States. Prehosp Disaster Med.

[11] Ari A, Fink JB (2012). Breath-actuated nebulizer versus small-volume nebulizer: efficacy, safety, and satisfaction. Respir Care.

[12] Azizkhani R, Hassan S, Boroumand A (2020). Analgesic effects of ketamine nebulizer vs. intravenous morphine in limb trauma patients in pre-hospital emergency setting; a randomized double-blinded clinical trial. Front Emerg Med.

[13] Jonkman K, Duma A, Velzen M (2017). Ketamine inhalation. Br J Anaesth.

[14] Jonkman K, Duma A, Olofsen E (2017). Pharmacokinetics and bioavailability of inhaled esketamine in healthy volunteers. Anesthesiology.

[15] Reddy M, Fiaz S (2017). Dose-dependent effectiveness of ketamine nebulization in preventing post-operative sore throat due to tracheal intubation. Sri Lankan J Anaesth.

